# Acupuncture in the treatment of fatigue in Parkinson's disease: A protocol for systematic review and meta-analysis

**DOI:** 10.1097/MD.0000000000023389

**Published:** 2020-11-25

**Authors:** Yingpeng Zhi, Chen Gao

**Affiliations:** Jinan Municipal Hospital of Traditional Chinese Medicine, Jinan, China.

**Keywords:** acupuncture, effectiveness, fatigue, parkinson's disease, protocol, systematic review

## Abstract

**Background:**

Fatigue is a commonly occurring nonmotor symptom among individuals of Parkinson's disease (PD). Little is known about how to measure fatigue in PD. This study is aiming to investigate the safety and efficacy of acupuncture for PD-related fatigue.

**Methods:**

RCTs of acupuncture for PD-related fatigue will be retrieved from inception to July 2020 in 9 different databases such as Cochrane Central Register of Controlled Trials, MEDLINE, EMBASE and so on. Search words will be used for the BC and acupuncture. The analysis would include randomised, controlled, clinical trials of PD patients with fatigue that were published in either Chinese or English. The primary outcome is the fatigue condition. Two or three reviewers should be in charge of study selection, data extraction and evaluating the risk of bias. RevMan software (V.5.3) will be used to perform the assessment of the risk of bias and data synthesis.

**Results:**

To provide evidence for the efficacy and safety of acupuncture treating PD-related fatigue.

**Conclusion:**

This study will be helpful for understanding the effect and safety of acupuncture for PD-related fatigue.

**Trial registration number:**

CRD42020160823

## 1 Introduction

Fatigue is a commonly occurring nonmotor symptom among patients diagnosed Parkinson's disease (PD).^[1,2]^ Even in the premotor phase of the disease, fatigue can be prevalent in PD patients.^[3]^ Once it manifests, it is most likely to continue or gradually become worse as the disease advances.^[4,5]^. Consequently, fatigue negatively impacts the life standard of PD patients.^[1,6]^ In has been reported that with 15.33% PD patients, fatigue was rated as the most disabling problem.^[7]^ Nevertheless, there is a lack of sufficient evidence to support the clinical treating of PD-related fatigue with drug or nondrug strategies.^[8,9]^

As a non-pharmacological treatment method, acupuncture provides a convenient and low-cost treatment method for effectively treating fatigue.^[10,11]^ There are several systematic reviews (SRs) on acupuncture for fatigue. As an example of the SR, consider Zhang et al's paper of acupuncture for chronic fatigue which was published in 2019, and included 16 randomized controlled trials (RCTs).^[10]^ This SR indicated that acupuncture was efficacy in alleviating chronic fatigue. The possible mechanism of acupuncture effect for fatigue may be the down-regulating of serum levels.^[12,13]^

Nowadays, the number of clinical reports on acupuncture for PD-related fatigue has gradually increased in the recent years.^[14–16]^ However, there is controversy surrounding the efficacy of using acupuncture for PD-related fatigue. There is a lack of SR and meta-analysis about the acupuncture for PD-related fatigue. Thus, there is a chance for us to conceive this SR to determine the efficacy and safety of acupuncture for PD-related fatigue.

## 2 Methods

**Figure 1:**
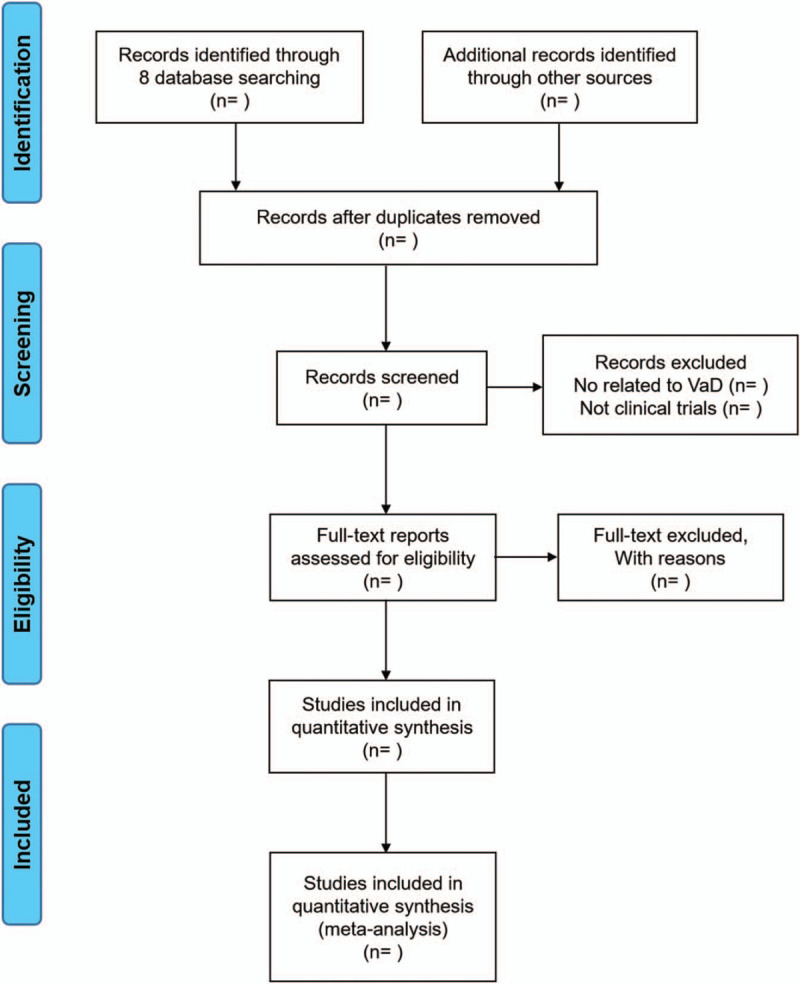
Flow diagram of studies identified.

This was registered on PROSPERO with CRD42020160823. The Preferred Reporting Items for Systematic Reviews and Meta-Analyses protocols (PRISMA-P) will be strictly followed (Fig. 1).^[17]^

### 2.1 Search strategy

Nine electronic databases, including 5 English databases (Cochrane Central Register of Controlled Trials, MEDLINE, EMBASE, Allied and Complementary Medicine Database, CINAHL) and 4 Chinese databases (China National Knowledge Infrastructure, Chinese Biomedical Literature Database, VIP Database, Wanfang Database) is going to be searched from inception to July 2020. The following terms: fatigue, Parkinson's disease, Parkinsonian disorders, acupuncture, manual acupuncture and electro-acupuncture, will be searched. The searching process for MEDLINE is listed in Table 1.

**Table 1:** Search strategy for the MEDLINE database.

Number	Search items
#1	randomized controlled trial [pt]
#2	controlled clinical trial [pt]
#3	randomized [tiab]
#4	placebo [tiab]
#5	clinical trials [MeSH]
#6	randomly [tiab]
#7	trial [ti]
#8	#1 OR #2 OR #3 OR #4 OR #5 OR #6 OR #7
#9	humans [MeSH]
#10	#8 and #9
#11	fatigue [tiab]
#12	Parkinson's disease [tiab]
#13	#11 and #12
#14	acupuncture therapy (MeSH)
#15	(acupuncture or body acupuncture or mamual acupuncture or electroacupuncture or electro-acupuncture): ti,ab
#16	#14 or #15
#17	#10 and #13 and #16

The following clinical trial registries will be used to retrieved ongoing trials: the NIH clinical registry ClinicalTrials.gov, the International Clinical Trials Registry Platform (ICTRP) and the Chinese clinical registry. We would retrieve the relevant SRs and meta-analyses manually and review it to identify additional studies. Useful but incomplete information would be acquired from the contact trial personnel.

### 2.2 Inclusion and exclusion criteria

#### 2.2.1 Types of study

RCTs reported in Chinese or English will be included. Others such as animal research, uncontrolled trials, or case reports will be excluded.

#### 2.2.2 Types of participants

Participants who diagnosed with PD based on UK Brain Bank criteria;^[18]^ and self-reported moderate or severe fatigue regardless of the criteria.

#### 2.2.3 Types of intervention

The acupuncture style considered must involve the insertion of needles at the acupuncture points. In addition, this study will include acupuncture therapy which is used alone or as an add-on to the conventional drug. At the same time, other types of acupuncture points without needle insertion into skin will be excluded. Studies comparing different acupoints or different acupuncture methods will be excluded.

#### 2.2.4 Types of control

The control group will be considered and classified as following: sham/minimal acupuncture, placebo, conventional therapies or routine care.

Studies will be excluded if the purpose of these is to compare acupuncture with other complementary therapies or different forms of acupuncture.

#### 2.2.5 Types of outcome measures

Fatigue condition is the primary outcome, which could be measured by the modified fatigue impact scale (MFIS). Secondary outcomes include the motor subsection of the Unified Parkinson's Disease Rating Scale (UPDRS), the Hospital Anxiety and Depression Scale (HADS), quality of life and adverse events. Quality of life related scales: the World Health Organization's Quality of Life Rating Scale (WHOQOL-100), the short-form 36(SF-36), the short-form (SF-12) and so on. Adverse events include two parts: the number of patients exiting and the number of patients reported adverse events.

### 2.3 Data collection and analysis

#### 2.3.1 Selection of studies

According to the inclusion criteria, all retrieved studies will be assessed by researchers (YPZ, CG) in the light of titles and abstracts. Full text of the qualified study will be reviewed if necessary. A third reviewer will arbitrate if any disagreement occurs. For excluded studies, the exclusion reason will be listed.

#### 2.3.2 Data extraction and management

Two reviewers (YPZ, CG) is going to double check and collect information from all qualifying studies and enter predefined data collection forms: author list and affiliation, publication source, country, interventions, outcomes, adverse effects and so on. The acupuncture intervention details will be elaborated according to STandards for Reporting Interventions in Clinical Trials of Acupuncture (STRICTA).^[19]^ A third reviewer will arbitrate if any discrepancy noticed. We would obtain the information which is not available by contacting the authors.

#### 2.3.3 Assessment of risk of bias in included studies

A systematic review of each study for the bias risk will be done by two or more independent reviewers by using the Cochrane Handbook for Systematic Reviews of Interventions.^[20]^ Six domains which are the bias of selection, performance, detection, attrition, reporting and other sources will be assessed. Trials is going to be rated as low risk, high risk, or unclear levels after evaluation. Involved authors will be contacted if anything unclear, discrepancies will be arbitrated by a third research as well.

### 2.4 Data synthesis

Data synthesis will be performed through RevMan software (V.5.3). Synthesize and analyze the data according to the level of statistical heterogeneity. The fixed-effects model will be used for the merged data if the detected statistical heterogeneity is small or low, otherwise, a random-effects model will be adopted. When necessary, the possible causes will be analyzed or a subgroup analysis will be conducted. If the heterogeneity is considerable in the included trials, no meta-analysis will be performed.

#### 2.4.1 Measures of treatment effect

Synthesizing and statistically analyzing of the power data will use RevMan V.5.3. The dichotomous data will be analyzed using a risk ratio with 95% CI. The mean difference or standard mean difference (SMD) with 95% CI will be used to analyze the continuous data. If different evaluation tools are used, SMD will be used.

#### 2.4.2 Management of missing data

Whether the data were intentionally missing or ‘randomly’ missing can be determined by contacting the corresponding author or author concerned. After the investigator concludes that the data are missing randomly, the available data will be analyzed. If not, it is necessary to apply to missing data to the trial's original investigator of the data or the contact listed in the trial registry. We will impute the missing data with replacement values in the event of no reaction from the authors or contact person, treating them as though they were observed. Missing values will be assumed using the last observation carried forward imputation method, followed by intention-to treat analysis. At the same time, if feasible, we will conduct a sensitivity analyses to address the potential impact of missing data. The potential impact of the effect of missing data on the final findings of the review will be addressed in the discussion.

#### 2.4.3 Assessment of heterogeneity

The forest plot survey will use the χ2 test with a significance level of *P* < .1 to investigat the statistical heterogeneity. At the same time, the inconsistency will be quantified by calculating the I^2^ test. The fixed-effect model will be used to pool the data. If there are heterogeneity tests in these trials that show little or no statistical heterogeneity (I^2^ < 50%). The random-effects model will be used for heterogeneous data (50% < I^2^ < 75%). The meta-analysis will not be conducted in the presence of great heterogeneity.

#### 2.4.4 Assessment of reporting biases

If there are more than ten studies included, funnel plots would be generated.

#### 2.4.5 Subgroup analysis

Subgroup analysis will be conducted if data is available. Variations will be considered in the characteristics of the treatments for acupuncture, participants, control types. And there will be subgroups to interpret the heterogeneity.

#### 2.4.6 Sensitivity analysis

The robustness of the main decisions made during the monitoring review process will be conducted through a sensitivity analysis. Several decision-making nodes for sensitivity review need to be considered in the system review process, such as methodological flaws, small research and data loss. Sensitivity analysis, as suggested in the Cochrane Handbook, includes two steps: in the first step, all major meta-analysis studies need to be included, and secondly, those studies that are known to be eligible. The results of the sensitivity analysis will be provided in the summary table. As shown by the results of the sensitivity analysis, the risk of bias will be discussed during the review process.

### 2.5 Grading the quality of evidence

The Grading of Recommendations Assessment, Development and Evaluation (GRADE) guidelines will be independently applied to measure the quality of outcomes.^[21]^ And the results will be present in “Summary of findings” tables. The assessments of evidence quality will be rated “high”, “moderate”, “low” or “very low”. Evidence of specific studies will be evaluated based on the risk of bias, inaccuracy, inconsistency, publication bias, indirectness, dose–response relation or effect size.

## 3 Discussion

PD has traditionally been characterized by progressive motor symptoms.^[22]^ Nonetheless numerous nonmotor symptoms are highly prevalent among patients with PD, such as fatigue, cognitive impairment, neuropsychiatric symptoms, and sleep dysfunction, some of which may precede the development of motor dysfunction.^[23,24]^ Nonmotor symptoms occur in all stages of PD, from prodromal, to early stages, to advanced disease, and can inform different PD subtypes with therapeutic and prognostic implications.^[25,26]^ Although the nonmotor symptoms is more detrimental to patients’ quality of life than the motor signs, they have not yet received equal attention in clinical and research. In recent years, common therapeutic methods such as anti-PD drug therapy do not consistently provide sufficient efficacy for PD related nonmotor symptoms, sometimes, the side effects of drugs may aggravate the motor symptoms of PD.^[27,28]^

In majority of the PD patients, symptoms of fatigue could be observed in the initial stage of the disease, and it is a persistent condition which can worsen as the disease advances.^[29,30]^ PD-related fatigue brings serious inconvenience to patients’ work and daily life, damaging their quality of life. At present, a satisfactory treatment method for PD-related fatigue has not been established. Acupuncture might be an effective therapy for PD-related fatigue.

Acupuncture as a non-pharmacological treatment is commonly considered a safe and effective treatment for a variety of conditions that cause discomfort.^[31]^ At the same time, acupuncture has many advantages such as, convenience, cost-effectiveness, less side effects, and it is also simple to use. As a result, patients widely accept the use of acupuncture. Although, the interpretation of acupuncture RCTs’ results are the subject of complex and controversy. The therapeutic effects of acupuncture treatment are likely to due to specific effects, nonspecific effects, trial-relevant effects and placebo effects. In view of the above factors, there are different opinions now. There is a view that it does not matter whether therapeutic effects of acupuncture are from placebo effects, given the relative safety of acupuncture.^[32]^ Others argue that a few researchers have found no specific effects of acupuncture and therefore against the use of acupuncture in clinical.^[33]^ We follow every patient's and doctor's point of view and draw their own conclusions. This SR will provide an assessment of the current state of acupuncture treatment for PD-related fatigue.

Useful conclusions would be drawn out from this SR may be helpful to patients with fatigue, clinical application specialist and decision makers. We have faith in the findings that it will be of great significance to both clinical practice and research. Four parts are in the course of processing this SR: study retrieval, study inclusion, data fetch and data synthesis. There may be some potential limitations with our study. First of all, the different forms of acupuncture may cause substantial heterogeneity. The second point further, studies which will be included in the future might have poor qualities, which will impose restrictions on the ability to come to conclusions based on high assurance.

## Author contributions

**Conceptualization:** Yingpeng Zhi.

**Data curation:** Yingpeng Zhi.

**Formal analysis:** Yingpeng Zhi.

**Funding acquisition:** Chen Gao.

**Investigation:** Yingpeng Zhi.

**Methodology:** Yingpeng Zhi.

**Project administration:** Chen Gao.

**Resources:** Chen Gao.

**Software:** Yingpeng Zhi, Chen Gao.

**Supervision:** Chen Gao.

**Validation:** Chen Gao.

**Visualization:** Chen Gao.

**Writing – original draft:** Yingpeng Zhi.

**Writing – review & editing:** Chen Gao.

## References

[1] Skorvanek M Nagyova I Rosenberger J Clinical determinants of primary and secondary fatigue in patients with Parkinson's disease. J Neurol 2013;260:1554–61. 23299623 10.1007/s00415-012-6828-4

[2] Fox SH Katzenschlager R Lim SY International Parkinson and movement disorder society evidence-based medicine review: Update on treatments for the motor symptoms of Parkinson's disease. Mov Disord 2018;33:1248–66. 29570866 10.1002/mds.27372

[3] Pont-Sunyer C Hotter A Gaig C The onset of nonmotor symptoms in Parkinson's disease (the ONSET PD study). Mov Disord 2015;30:229–37. 25449044 10.1002/mds.26077

[4] Friedman JH Friedman H Fatigue in Parkinson's disease: a nine-year follow-up. Mov Disord 2001;16:1120–2. 11748745 10.1002/mds.1201

[5] Alves G Wentzel-Larsen T Larsen JP Is fatigue an independent and persistent symptom in patients with Parkinson disease? Neurology 2004;63:1908–11. 15557510 10.1212/01.wnl.0000144277.06917.cc

[6] Stocchi F Abbruzzese G Ceravolo R Prevalence of fatigue in Parkinson disease and its clinical correlates. Neurology 2014;83:215–20. 24928125 10.1212/WNL.0000000000000587

[7] Havlikova E Rosenberger J Nagyova I Impact of fatigue on quality of life in patients with Parkinson's disease. Eur J Neurol 2008;15:475–80. 18325024 10.1111/j.1468-1331.2008.02103.x

[8] Weintraub D Burn DJ Parkinson's disease: the quintessential neuropsychiatric disorder. Mov Disord 2011;26:1022–31. 21626547 10.1002/mds.23664 PMC3513835

[9] Franssen M Winward C Collett J Interventions for fatigue in Parkinson's disease: a systematic review and meta-analysis. Mov Disord 2014;29:1675–8. 25234443 10.1002/mds.26030

[10] Zhang Q Gong J Dong H Acupuncture for chronic fatigue syndrome: a systematic review and meta-analysis. Acupunct Med 2019;37:211–22. 31204859 10.1136/acupmed-2017-011582

[11] Zhang Y Lin L Li H Effects of acupuncture on cancer-related fatigue: a meta-analysis. Support Care Cancer 2018;26:415–25. 29128952 10.1007/s00520-017-3955-6

[12] Qing P Zhao JF Zhao CH Effect of acupuncture on patients with cancer-related fatigue and serum levels of CRP, IL-6, TNF-α and sTNF-R. Zhongguo Zhen Jiu 2020;40:505. 32394658 10.13703/j.0255-2930.20190423-k0002

[13] Shui L Yi RN Wu YJ Effects of mongolian warm acupuncture on iNOS/NO and inflammatory cytokines in the hippocampus of chronic fatigue rats. Front Integr Neurosci 2020;13:78. 32082125 10.3389/fnint.2019.00078 PMC7006054

[14] Kluger BM Rakowski D Christian M Randomized, controlled trial of acupuncture for fatigue in Parkinson's Disease. Mov Disord 2016;31:1027–32. 27028133 10.1002/mds.26597

[15] Kong KH Ng HL Li W Acupuncture in the treatment of fatigue in Parkinson's disease: A pilot, randomized, controlled, study. Brain Behav 2017;8:e00897. 29568693 10.1002/brb3.897 PMC5853635

[16] Guangzhou University of Chinese Medicine, Lin YY Clinical study on the treatment of parkinson's disease using Du meridian activating acupuncture therapy combined with three-needle trembling therapy[D]. 2018.

[17] Shamseer L Moher D Clarke M Preferred reporting items for systematic review and meta-analysis protocols (PRISMA-P) 2015: elaboration and explanation. BMJ 2015;4:1–9. 10.1186/2046-4053-4-1 PMC4320440 25554246

[18] Hughes AJ Daniel SE Kilford L Accuracy of clinical diagnosis of idiopathic Parkinson's disease: a clinico-pathological study of 100 cases. J Neurol Neurosurg Psychiatry 1992;55:181–4. 1564476 10.1136/jnnp.55.3.181 PMC1014720

[19] Macpherson H Altman DG Hammerschlag R Revised STandards for Reporting Interventions in Clinical Trials of Acupuncture (STRICTA): extending the CONSORT Statement. J Evid Based Med 2010;3:140–55. 21349059 10.1111/j.1756-5391.2010.01086.x

[20] Wang S Yang H Zhang J Effcacy and safety assessment of acupuncture and nimodipine to treat mild cognitive impairment after cerebral infarction: a randomized controlled trial. BMC Complement Altern Med 2016;16:361. 27623621 10.1186/s12906-016-1337-0 PMC5022140

[21] Guyatt GH Oxman AD Vist GE GRADE: an emerging consensus on rating quality of evidence and strength of recommendations. BMJ 2008;336:924–6. 18436948 10.1136/bmj.39489.470347.AD PMC2335261

[22] Kalia LV Lang AE Parkinson's disease. Lancet 2015;386:896–912. 25904081 10.1016/S0140-6736(14)61393-3

[23] Schapira AHV Chaudhuri KR Jenner P Non-motor features of Parkinson disease. Nat Rev Neurosci 2017;18:435–50. 28592904 10.1038/nrn.2017.62

[24] Goldman JG Guerra CM Treatment of Nonmotor Symptoms Associated with Parkinson Disease. Neurol Clin 2020;38:269–92. 32279710 10.1016/j.ncl.2019.12.003

[25] Fereshtehnejad SM Postuma RB Subtypes of Parkinson's disease: what do they tell us about disease progression? Curr Neurol Neurosci Rep 2017;17:34. 28324303 10.1007/s11910-017-0738-x

[26] Konno T Al-Shaikh RH Deutschlander AB Biomarkers of nonmotor symptoms in Parkinson's disease. Int Rev Neurobiol 2017;133:259–89. 28802922 10.1016/bs.irn.2017.05.020

[27] Ziemssen T Reichmann H Non-motor dysfunction in Parkinson's disease. Parkinsonism Relat Disord 2007;13:323–32. 17349813 10.1016/j.parkreldis.2006.12.014

[28] Schaafsma JD Balash Y Gurevich T Characterization of freezing of gait subtypes and the response of each to levodopa in Parkinson's disease. Eur J Neurol 2003;10:391–8. 12823491 10.1046/j.1468-1331.2003.00611.x

[29] Valko PO Waldvogel D Weller M Fatigue and excessive daytime sleepiness in idiopathic Parkinson s disease differently correlate with motor symptoms, depression and dopaminergic treatment. Eur J Neurol 2010;17:1428–36. 20491889 10.1111/j.1468-1331.2010.03063.x

[30] Herlofson K Kluger BM Fatigue in Parkinson's disease. J Neurol Sci 2017;374:38–41. 28087059 10.1016/j.jns.2016.12.061

[31] World Health Organization, World Health Organization Acupuncture: review and analysis of reports on controlled clinical trials[M]. 2002.

[32] Musial F Acupuncture for the treatment of pain–a mega-placebo?[J]. Frontiers in Neuroscience 2019;13:1110. 31680841 10.3389/fnins.2019.01110 PMC6811493

[33] Colquhoun D Novella SP Acupuncture is theatrical placebo. Anesth Analg 2013;116:1360–3. 23709076 10.1213/ANE.0b013e31828f2d5e

